# Little women: the relevance and reliance on mouse models for mammary gland research and next steps for translation

**DOI:** 10.1007/s10911-025-09590-8

**Published:** 2025-11-20

**Authors:** Laura B. Bjerre, Silke B. Chalmers, Felicity M. Davis

**Affiliations:** 1 Department of Biomedicine, Aarhus University, Aarhus, Denmark; 2 Danish Research Institute of Translational Neuroscience, Aarhus University, Aarhus, Denmark; 3 School of Biomedical Sciences, University of New South Wales, Sydney, Australia; 4 School of Pharmacy, University of Queensland, Brisbane, Australia

**Keywords:** Mammary Gland, Development, Puberty, Lactation, Involution, Mouse Models

## Abstract

The neglect of research into women’s health and female biology has had major impacts for the fields of mammary biology and cancer. A quarter of the way through the twenty-first century, we still lack basic knowledge regarding the formation and function of the organ that gives its name to all mammals, and which provides important health benefits for children and their breastfeeding parent through the creation and delivery of breast milk. In this review, we highlight key similarities and differences in mouse and human mammary glands, and discuss how both systems of investigation are important and necessary to fill outstanding knowledge gaps. We discuss important discoveries that have arisen through mouse models as well as methodological advances that have enabled more widespread investigations in human samples. Finally, we contend that the translatability of mammary gland research requires thoughtful design, careful evaluation and continued review, irrespective of the system of investigation.

## Introduction

### The state of the field – mammary gland development and physiology

In many fields of the life sciences, there has been a growing push—both from funders and research institutes—toward translation and application [1]. Whilst any ambition to improve human health is commendable, a unidimensional focus on translation neglects the basic human quality of curiosity (including curiosity about how our own bodies develop and function) and fails to acknowledge that major health advances are often born in “basic science” (herein referred to as “foundational science”) laboratories, often serendipitously [1, 2]. Weighted priorities applied to already scarce funding resources (e.g., the recent prioritization by the NIH for “human-based research technologies” [3], as well as known biases and questionable reliability in the peer review process, stifles innovation and discovery [4]. Another challenge exists, however, for neglected fields of research, including the field of female biology and women’s health research [5, 6]. In these cases, we often lack a fundamental understanding about development, physiology and mechanisms of disease, broadening the gap between what we know and what we need to know to expedite translation and application.

Much remains unknown in the field of mammary gland development and physiology, despite the central role of this organ in mammalian health and survival [7]. The mammary gland (or breast in humans) is a fascinating organ, which begins developing in the embryo but undergoes major phases of expansion and remodeling in the adult [8]. In its mature stage, it produces a fluid that acts to sustain the next generation, the benefits of which are only just beginning to be understood through large longitudinal studies [9]. But for some individuals, it is an organ that may never serve a purpose and one that is, in fact, prone to disease (e.g., breast cancer), physical discomfort (e.g., back pain necessitating breast reduction surgery) or even the cause of psychological distress (e.g., as a result of unrealistic beauty standards). A greater understanding of breast biology is urgently needed, not only for the ~ 85% of people who bear children and intend/ed for them to be breastfed [10], but for all people susceptible to breast cancer. It comes as no surprise that our ability to design new treatments for breast cancer is limited by our general lack of understanding of breast epithelial cells and their stromal counterparts [11, 12]. Similarly, our ability to regenerate breast tissue, e.g., for reconstruction post-mastectomy, relies on our understanding of breast stem cells and their mechanisms of regulation.

**Fig. 1:**
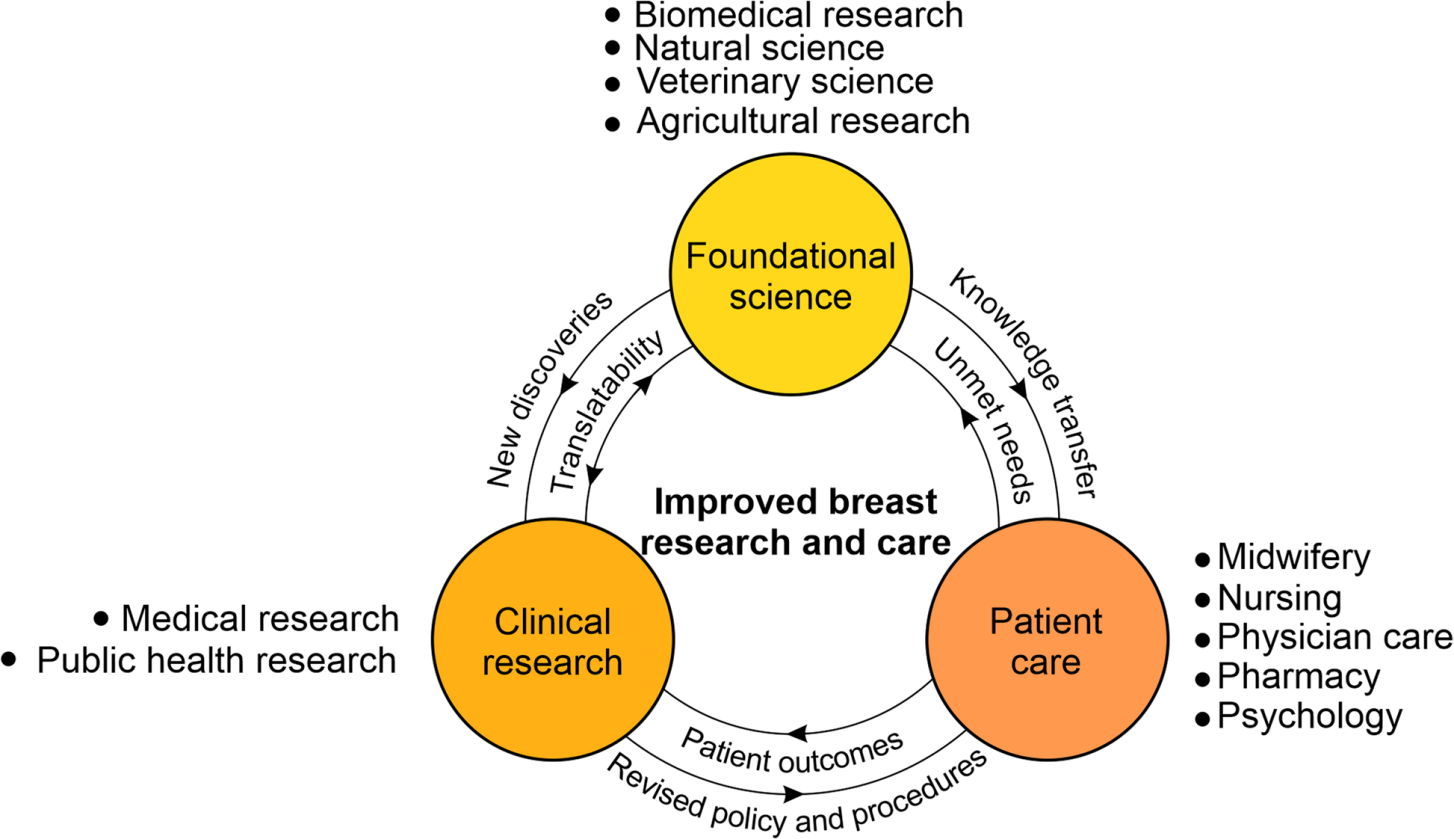
Improvements in breast research and patient care require continual communication and collaboration across the domains of foundational and clinical research and patient care. Foundational science includes, but is not limited to biomedical, natural, veterinary and agricultural research. Clinical research includes medical research and public health research. Patient care involves the professions of midwifery, nursing, physician care, pharmacy, psychology and others. A greater focus on improved communication between foundational researchers and patients and their carers is increasingly recognized as important

Knowledge gain in this space requires communication and collaboration between clinical researchers (studying human breast physiology and pathology), public health researchers (studying long term outcomes of breastfeeding), foundational scientists (using model organisms—such as mice—to understand mammary biology and disease) and veterinary researchers (using domestic and agricultural mammals that may more closely resemble some aspects of human biology), as well as engagement with physicists and computational scientists. It also requires a circulation of knowledge from the laboratories to the clinic and back again, to ensure that both knowledge and knowledge gaps are shared and understood (Fig. 1).

In this review, we discuss the relevance and enduring importance of mouse models for mammary gland research, pointing to research topics where studies in humans have been severely limited by a dearth of available and relevant material or by technical challenges. This review is not intended to draw comparisons between all major studies utilizing mouse and human tissue; rather, it provides some examples where studies in human are optimal and increasingly feasible, and discusses how novel technologies and research tools applied to samples of both species may help to shine new light on this important area of research. We apologize to the authors of the works that have been omitted from this review.

### The mouse mammary gland: Similarities and differences to the human breast

Like the human breast, the mouse mammary gland consists of a bilayered epithelium that is made up of an inner layer of polarized luminal cells surrounded by basal (or myoepithelial) cells [13]. Mammary ducts branch through the adipocyte-rich stroma, which is also populated by immune, endothelial, nerve and fibroblast cell types. The glands of female mice and humans undergo comparable developmental milestones, including key stages in the embryo and at puberty, gestation, lactation as well as post-lactational and age-related involution [13], each of which are a focus of discussion later in this review. Although there are differences between the rodent and human hormonal milieu [14], both species have cyclic estrus/menstrual phases (with some proliferative heterogeneity observed in mouse [15, 16] and typically more than one pregnancy over their reproductive lifetime [17]. Thus, their mammaries are subject to recurring patterns of cell proliferation and cell death.

**Fig. 2:**
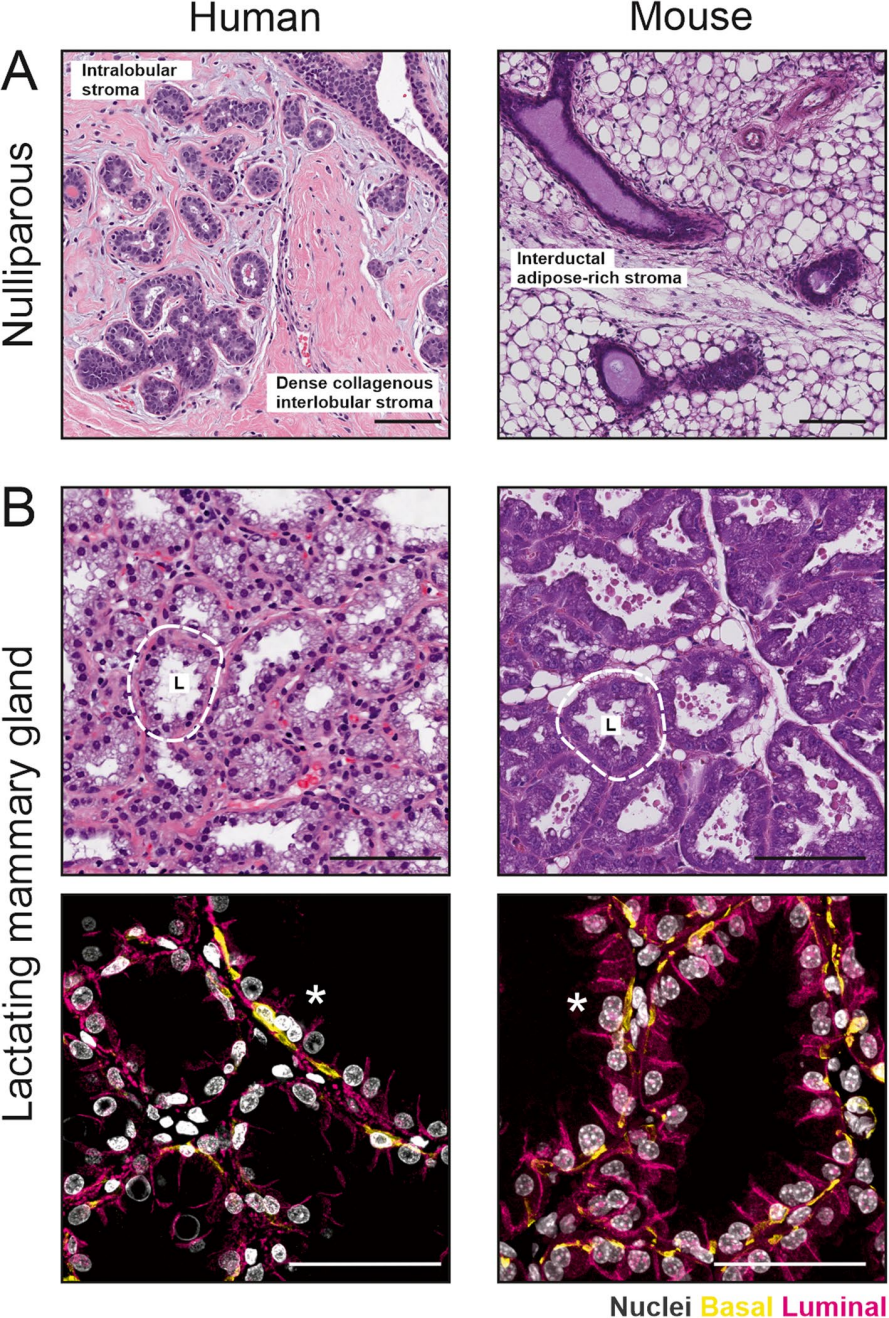
Morphological comparison of human and mouse mammary glands. **A** Nulliparous mammary gland structures are embedded within a collagen- or adipose-rich stroma in human and mouse, respectively. **B** The lactating mammary gland of human and mice are organized into milk producing alveolar units (outlined) where milk is secreted into the central alveolar lumen (L). Immunohistochemical staining shows basal cells (yellow) surrounding luminal cells (examples of binucleated cells highlighted with asterisk) bound together by adherens junctions (magenta). Nuclei are stained with DAPI (gray). Scale bars are 100 µm and 50 µm for H&E and IHC images, respectively

Notable differences exist between mouse and human mammary glands, which have been reviewed in-depth in dedicated reviews in this journal and others [13, 14, 18]. The most obvious difference is mammae number—one pair in humans and five pairs in mice [13]. This presents an anatomical limitation on simultaneous nursing ability but does not strictly cap a lactating parent’s maximum nursing ability [19]. Milk composition also varies between mouse and human, including many key components such as calcium [20], fat and protein [21, 22], reflecting the speed of neonatal development and duration of postnatal care. The organization of ductal structures differs between mouse and human, with mice having a single nipple-draining duct and humans having multiple collecting ducts draining to the nipple [13]. Human tissue is also arranged into terminal ductal lobular units (TDLUs) and has a “specialized stroma”, which is not present in mice (Fig. 2) [13, 23]. This consists of “loose” collagen and other matrix proteins that surround the TDLU. A more dense interlobular stroma surrounds each TDLU structure (Fig. 2). The stroma of the mouse mammary gland, on the other hand, is mainly composed of adipose tissue. Stromal cells are positioned around ductal structures and throughout the mammary fat pad.

**Fig. 3:**
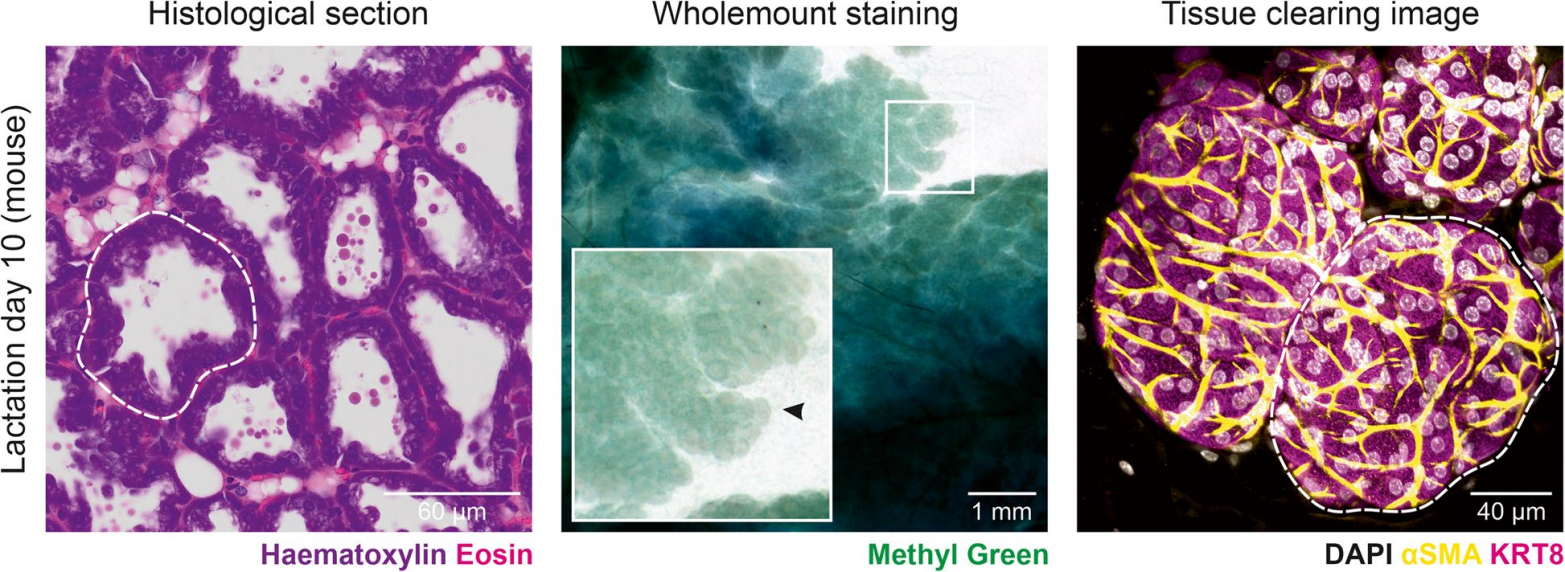
Multidimensional imaging of lactating mouse mammary gland. **A** Micrograph showing a ~ 5 µm thick section cut from a formalin-fixed paraffin-embedded block, which has been stained with haematoxylin and eosin. **B** Image showing a mammary gland wholemount stained with methyl green (middle). **C** Confocal micrograph showing a maximum intensity projection of ~ 70 µm (*z*) of cleared mammary tissue immunostained for keratin 8 (magenta), alpha-smooth muscle actin (yellow), and with DAPI staining in gray (right). An alveolar unit is circled in **A** and **C** and marked by an arrowhead in **B**

Over the last decade, spectacular 3-dimensional (3D) images of mouse mammary gland architecture have continued to emerge and have even become commonplace in many studies, overcoming many of the limitations of extrapolating glandular structure from flat histological sections and macroscopic wholemount images [24–28] (Fig. 3). These high-resolution 3D images are helping us to appreciate the true cellular organization of this organ at different developmental stages. Similar studies in human tissue, however, are lagging. A recent manuscript exploring TDLU structures in 3D in tissue obtained from breast reduction surgeries or “normal” tumor-adjacent tissue from mastectomies has revealed intriguing heterogeneity in cytokeratin (KRT) 14 expression in basal cells of the main subtree versus the remaining TDLU structure, demonstrating additional levels of organization and providing a means of identifying major branches [29]. This manuscript also revealed that branching morphogenesis of TDLUs appears to follow a volume-limited 3D branching and annihilating random walk model, not dissimilar to principles governing mammary branching morphogenesis in mice. Whether these expression patterns change during pregnancy and active breastfeeding, when the gland remodels and basal cells assume the important task of milk ejection, however, remains to be seen. Volumetric imaging of the human and mouse mammary glands during lactation have helped to confirm the presence of binuclear cells in the mammary gland, with approx. 30% of secretory cells in the lactating human breast having two nuclei (cf approx. 50% in mouse) [30]. Perhaps it is also important to note here that differences exist not only between human and mouse tissue, but between mouse strains, (e.g., 129 and C57BL/6 J mice [31, 32]), including strain-specific variations side branching in the topology and the gland, highlighting the need for rigorous and transparent methods, regardless of the system of investigation.

Mouse mammary glands have noteworthy differences to their human counterparts, limiting their utility in certain research contexts [13]. However, owing to their considerable similarities with human breast tissue; their amenability to genetic manipulation; reduced confounding variables (e.g., hormone supplementation and parity); their general superiority over propagated cells and cell lines; and their availability to nearly all research laboratories around the world, they remain both important and necessary for knowledge gain in breast biology and women’s health research [33]. In this review, we journey through the phases of mammary gland development—from embryo to age-related involution—discussing key lessons learned from mice, as well as insights gained from the judicious use of often hard-to-come-by human samples. We direct the majority of our discussion to mammary biology and physiology (rather than mammary cancer models), noting that laboratory mice seldom develop spontaneous tumors. We direct the reader to recent reviews discussing the utility of other model systems that can be used to study tumor development, where relevant, most notably unneutered cats and rabbits [34].

## Embryo

Studies exploring prenatal mammary gland development offer fascinating insights into both developmental and cancer programs [35–39]. However, owing to obvious ethical and logistical challenges, studies have predominately been conducted in model organisms, most notably mouse and rabbit, the latter of which is more appropriate for modelling human male mammary gland development [40–42]. Rare glimpses of human fetal breast tissue, however, have been obtained [43–45], including micrographs by Howard and Gusterson in this journal more than 25 years ago [46]. These intriguing images highlight important structural similarities between embryonic mouse and human mammary glands, supporting the use of mouse models in this field of research. An important observation from this work, however, was the clear lack of predictability in chronology of early human breast development (cf mice), which the authors hypothesize is due to individual variation in hormone levels, both in utero and post-birth. To overcome this, staging of the prenatal human breast may be more accurately determined based on the length of the embryo [46, 47].

Insights from humans have largely been garnered from the study of rare syndromes associated with the absence of breast and nipple tissue or their supernumerary [46]. For example, ulnar-mammary syndrome, which can affect limb, apocrine gland, tooth, genital and breast development in humans, is known to be caused by mutations to *TBX3*. Development of mouse mutants of *Tbx3* have confirmed failure of mammary bud induction [48] and expression analysis in mouse embryos and creation of overexpression models point to roles for *Tbx3* and *Bmp4* in mammary line specification [38, 49]. For a list of human syndromes linked to embryonic development see Ref [38]. Howard and Gusterson also note in their seminal study [46] that the presence of a milk line or crest in humans is supported in theory by cases of supernumerary nipples, which are found from the groin to the axilla. Further support for this idea came from their samples, some of which showed a thickening extending across the embryo that was associated with condensation of epithelial cells in the thoracic/pectoral region, where the breast bud typically forms.

Compared to humans, characterization of embryonic mammary development is unquestionably easier in mice. However, technical challenges, most notably related to the small size of mouse mammary buds and branches in mid- and late-stage embryos, make studies during this developmental window difficult and thus less frequent than postnatal investigations. Nevertheless, our knowledge of mouse prenatal mammary gland development has benefited from advances and refinements in the isolation and culture of embryonic mammary structures in their native stroma [50–52]. Using these protocols, combined with state-of-the-art microscopy, fresh insights into mammary branch patterning and branch point frequency have been obtained, helping to shed light on mechanisms of prenatal branching morphogenesis in an organ with a relatively “sparse” ductal network (cf. kidney and lung) [35]. Back-to-back studies in 2018, using mammary rudiments dissected from specific fluorescent reporter models, have revealed early lineage-restriction of mammary epithelial cells [36, 37], and subsequent work helped to elucidate the spatiotemporal signals that guide embryonic mammary cell fate specification [53]. These studies and others have provided invaluable new knowledge about branching morphogenesis, lineage commitment and the early creation of the structures which lend their name to the entire class of mammals. The availability of shared technical resources is a major advantage to the field. Future studies characterizing embryonic mammary structures of other species and (when possible) human tissues will continue to shed light on this important field of research.

## Puberty and maturity

As is the case for embryonic research, challenges and limitations with human sample collection during puberty mean that much of our knowledge about this stage of development originates in mice, where research teams are actively investigating mechanisms of branching morphogenesis and cell lineage relationships [25, 54, 55], reviewed recently in Ref [8] and discussed in more detail below. An obvious exception are studies examining breast density and the influences of circulating hormones, where valuable insights can be obtained from human subjects using relatively non-invasive imaging modalities and blood sampling, respectively [56]. Using this approach, a link has been made between adult breast density and premenarcheal dehydroepiandrosterone sulfate (DHEAS) and sex hormone-binding globulin (SHBG) [57], although the role of SHBG remains contentious (summarized in Ref [56]).

The availability of excess tissue obtained from reduction mammoplasties and gender affirming surgeries makes studies in the mature adult breast more common. Whilst breast reduction and removal procedures can provide researchers around the world with large volumes of sample and are a valued resource, a study comparing histological profiles of tissue derived from these procedures has demonstrated that only 12% of reduction mammoplasty samples are histologically normal (cf. 65% of donor biopsies examined from the Susan G Komen Tissue Bank) [58]. Although this study has its limitations, particularly those associated with the method and volume of tissue obtained, issues related to the use of these tissues (as well as tumor-adjacent tissue [59] or tissue derived from prophylactic mastectomy [60] or autopsy [61]) as a means for studying processes characteristic of the normal (non-diseased) breast should be discussed in manuscripts, ideally coupled with histopathological review and guided sample selection [62].

The categorization of some human cell lines as “normal” is also a source of potential confusion for in vitro breast research. The MCF10 cell lines, first described by Soule et al. in a pioneering publication that has now been cited more than 2000 times [63], are used in a diverse range of assays, including studies examining normal 3D cellular organization and polarity [64], studies elucidating the genetic drivers of malignancy [65, 66] and as a point-of-reference in many studies examining characteristics of cancer cell lines [67]. Whilst their use has enabled many discoveries, it is important to reflect that these cells were obtained from a 36-year-old parous woman undergoing mastectomy who was diagnosed with extensive fibrocystic breast disease [63]. Seeking to widen the pool of available normal breast-derived epithelial cells, well characterized cell lines derived from core biopsies have now been established by some groups [68]. Non-immortalized human mammary epithelial cells (HMECs) offer a time-limited alternative to use of immortalized cells and are commercially available through numerous vendors. An important consideration related to the use of any cultivated human cell lines, however, is a lack of models representing hormone receptor positive populations. As reviewed by Brisken and Scabia [69], this is likely caused by a loss of hormone receptor expression in culture and a drift toward cells with a basal phenotype.

Despite challenges, primary cells from human breast tissue have been used for the creation of 3D organoid systems, offering other opportunities to investigate aspects human breast biology in vitro, including questions related to cellular organization and differentiation states [62, 70–73]. These cultures have been derived from diverse starting materials, ranging from single cells and cell clusters to minced tissue fragments. A study from the Brugge laboratory in 2020 demonstrated that human breast cells (obtained from tissue confirmed to be histologically normal) could form organoids in basement membrane extract (BME), which could be propagated for more than 16 months in culture and which retained protein expression patterns resembling the fresh tissue that it was derived from [62]. This was in contrast to cells propagated in 2D, where luminal cells were largely lost and where the remaining cells adopted an abnormal protein expression pattern [62]. Thus, whilst organoid models are not without flaws, they offer additional avenues for assessing human and mouse biology in a more physiological setting than cells grown on plastic.

Mouse models remain relevant and important for studying many aspects of mammary gland development and homeostasis, including mammary stem and progenitor cell dynamics, which is optimally studied in vivo [74, 75]. Using transgenic mouse models expressing inducible reporter genes it has been demonstrated that luminal and basal cell lineages are specified in the embryo [36, 37, 76] and that under physiological conditions the postnatal mammary gland is formed by the action of lineage-restricted cells [54, 76–80]. Using similar fluorescent reporter-based lineage tracing strategies, however, some groups have found that a population of basal cells can give rise to both luminal and basal progeny [24, 81]. Whilst it is now widely agreed that mammary cell transplantation or “wounding” unleashes bipotency in the basal compartment [82, 83], it remains uncertain how to best reconcile opposing observations from experiments that were performed under physiological (non-transplant) conditions. These discrepancies remind us of the importance of investing in diverse research teams to simultaneously address important research questions and the need for thoughtful discussion and analysis of disparate findings. It speaks to having a critical mass of researchers in each (sub)field of research, a goal that can be hard to achieve in neglected areas of research. The uptake of technologies to genetically barcode cells and analyze cellular ancestries also permits investigations of lineage relationships in mice for developmental and cancer research [84]. Lineage tracing of “natural” barcodes present in human cells also opens the door for retrospective lineage analysis in non-diseased human breast samples [75, 85], which is an exciting topic of future investigation. Early applications of this approach in human breast cancers, including a discussion of its caveats can be found in Ref [75].

## Pregnancy

During pregnancy, the mammalian body undergoes profound changes, comparable to no other physiological stage of adult life. These changes include alterations in hormonal profiles [86], metabolic rate [87], cardiovascular function [88] and neuroanatomical structure [89–91], which must be precisely coordinated to support both fetal development and the necessary transition to postnatal care. The mammary glands are no exception. To prepare for lactation, the mammary epithelium undergoes extensive expansion and functional differentiation, a process known as alveologenesis [74]. These changes are primarily driven by key hormonal regulators, which orchestrate alveologenesis through a complex interplay of endocrine and paracrine signals. This glandular remodeling has recently been reviewed elsewhere [92]. Here, we discuss clinical research exploring the benefits of colostrum (the first milk) for human health and draw on limited mechanistic studies—largely from the agricultural sciences—exploring mechanisms of colostrogenesis.

Colostrum is a thick, yellow fluid that can leak or be expressed antenatally and continues to be produced several days postpartum [93]. It is enriched with immunological components, including immunoglobulin A, lactoferrin, immune cells and cytokines [94, 95]. These factors are crucial for immune protection and maturation of the infant’s naïve immune system. Colostrum intake is associated with improved infant survival and is directly linked to reduced cases of necrotizing enterocolitis [96]. Evidence links early initiation of colostrum intake to reduced infant infections and mortality, which has helped to inform the Baby-Friendly Hospital Initiative (BFHI) [97] and the WHO’s recommendation to start breastfeeding within the first hour [98].

Antenatal colostrum expression, or colostrum “harvesting”, is gaining attention. While some agencies recommend postponing to weeks 36–37, due to the theoretical risk of uterine contractions [99–101], a large-scale randomized controlled trial demonstrated that antenatal colostrum expression in low risk, diabetic, singleton pregnancies from week 36 did not increase risks of preterm birth or neonatal intensive care unit admission [102]. The authors of this study recommend that low-risk diabetic patients express milk late in the third trimester to improve the likelihood of newborns receiving breast milk exclusively on the first day of life. The beneficial effects of colostrum in stabilizing blood glucose may have additional benefits for these children, who are at higher risk of neonatal hypoglycemia [102]. Additional qualitative studies suggest that this practice may also improve breastfeeding confidence and familiarity [103–105]. Moreover, a pilot study of healthy pregnancies where colostrum was harvested from 34 weeks, showed no difference in gestational age at birth and no adverse effects [106]. However, whilst milk was available to more than 80% of the intervention (expressing) group, no statistical difference in breastfeeding outcomes was observed. More research is needed to understand the full risk–benefit profile in the general population to inform and update clinical guidelines.

Although some expecting parents can collect colostrum during pregnancy, the biological determinants of this capacity and the timing of readiness of the gland remain poorly defined, particularly in relation to parity, multiple pregnancy and medical complications. Given the health benefits of colostrum and early initiation of breastfeeding, there is a need for mechanistic studies elucidating the pathways that guide colostrogenesis and alveologenesis in humans. This work has the potential to inspire innovative support structures for expectant parents eager to breastfeed. While mouse models remain invaluable for some studies, their relevance for colostrum research might be limited [107]. Mice primarily transfer immunoglobulins through the placenta during gestation, suggesting that the first milk might play only a minor role for neonatal immune protection in this species [107]. To advance our understanding of colostrum production and secretory activation, there is a need for more investigations in non-traditional model organisms with physiological and immunological similarities to human. Species such as sheep, pigs and cows are particularly relevant in this respect, with the obvious advantage of improved sample availability compared to humans [34, 107].

## Breastfeeding and lactation

Studies exploring human breast tissue during lactation are limited by a range of factors, including availability of donor tissue; consistency of sample collection and processing; and variability in breastfeeding factors and donor history [12]. Despite substantial challenges, much can still be learned by the careful collection, curation and analysis of human milk samples, as well as the monitoring of outcomes for the breastfeeding dyad in longitudinal studies.

Breast milk is now described as the “first personalized medicine”, as its composition is tailored to meet the infant’s developmental stage and precise immunologic and metabolic requirements [108]. *How* the mammary gland fine-tunes the composition of the fluid it is creating for another being, however, is a fascinating topic of research and one that remains the subject of continued investigation. It is also unclear exactly what this means for parents of multiples (e.g., twins, particularly dizygotic twins), those performing tandem breastfeeding (that is, the breastfeeding of two children of different ages at the same time) and parents who engage in milk sharing. This population complexity—further confounded by factors including breastfeeding history, total duration of breastfeeding, duration of exclusive breastfeeding, the use of bottles to deliver breast milk and maternal health and wellbeing—can be difficult to capture and account for. Nevertheless, its due recognition within the domain of personalized/precision medicine, together with a growing interest in microbiome research, has helped to reframe lactation research from something formerly considered “not modern” or primarily relevant to the dairy industry to something increasingly seen as interesting and impactful [108]. The value of breastfeeding, to both human health and economy, has been comprehensively reviewed in two dedicated series in The Lancet from 2016 and 2023 [109, 110]. Understanding the “non-nutritive functions of milk (including protection against infection, promotion of intestinal, immune and cognitive development etc.) has been set as a research priority for the field for over a decade and remains and area of active investigation. Prominent studies seeking to understand mechanisms underpinning the health/wellbeing benefits of milk have been performed in both human patients and mice, depending on feasibility and relevance. For example, an important study examining the abundance of colonic ROR-gt^+^ regulatory T cells in offspring, observed variations in proportions of ROR-gt^+^ cells among FoxP3^+^ regulatory T cells between inbred mouse strains, and utilized this phenomenon to examine modes of multi-generational transmission by strategic intercrossing [111]. On the other hand, an important mechanistic study examining the impact of breast milk on offspring IQ utilized existing human cohort data [112].

A major factor contributing to the personalization of breast milk relates to its living cellular component, of both bacterial and host origin [113]. Human breast milk contains a heterogenous population of host cells, the most well-known and studied in early stage milk being immune cells [114]. Remarkably, milk also contains substantial numbers of mammary epithelial cells, which are shed into the alveolar lumen during the process of milk production, and which remain viable after milk ejection [115]. Recent advances in single cell transcriptomic profiling have helped to uncover the breadth of host cells present within milk, including the identification of at least two transcriptionally distinct luminal epithelial subtypes [115]. In addition, up to seven immune cell types have been characterized, with macrophages representing the most abundant population [116]. Although it was previously believed that immune cell levels stabilized and remained constant after peaking during early lactation, evidence now strongly suggests that milk immunity is more dynamic and responsive to external cues, particularly infant infection [116–120]. The immune responsiveness of milk during infant infection has been hypothesized to be triggered by either simultaneous systemic infection of the lactating parent or through retrograde flow of infant-derived antigens from saliva through the nipple during breastfeeding, however, direct evidence of this latter phenomenon is difficult and lacking [121]. Our understanding of milk immune modulation was bolstered by the COVID-19 pandemic, where studies confirmed that vaccination of the lactating parent enhanced the immune component of their milk, potentially offering increased protection to the child during periods of elevated risk [122].

Whilst studies exploring the breastfeeding dyad continue to shed light on mechanisms of milk personalization, research within this field also highlights widespread trends in volume and composition changes through lactation. Broadly, milk volume increases dramatically in the early postpartum period, and continues to rise gradually alongside infant growth during the first months of life, although exact volumes are difficult to quantify and exhibit variability both within and across cohorts (e.g. parous vs first time parents, singleton vs parents of multiples) [123–126]. The composition of milk also evolves with time, with colostrum being enriched with immune factors and mature milk providing a high level of nutritional support for growth [94, 107]. Milk composition varies not only across the weeks and months of lactation but also within a 24-h cycle. Circadian variation in both macro- and micro-nutrient content, as well as in hormonal concentrations have been observed [127], with levels of amino acids and minerals being higher in daytime feeds. Night milk, on the other hand, is enriched in nucleotides such as guanylic acid and adenylic acid, which are thought to be associated with the release of sleep-promoting hormones, as well as in melatonin and fat, which may contribute to improved infant sleep [127]. In contrast, cortisol concentrations in morning milk have been shown to be significantly higher than afternoon and evening milk, potentially promoting infant alertness and supporting daytime behavior regulation [127, 128]. It is also worth noting that breast milk composition changes over the course of a single feed, with foremilk being thinner than hindmilk, potentially enabling immediate hydration and subsequent satiety [129]. An important recent review from the Ingman group, however, highlights the pre-analytical and analytical challenges of breast milk analysis, including timing of sample collection, sample storage requirements and the effect of the entire solution composition on the measurement of each component being analyzed [130].

For the nursing parent, breastfeeding reduces the overall breast cancer risk and improves birth spacing, with implications for ovarian cancer and type 2 diabetes [9]. Its role in breast cancer protection, however, is complex and dependent on the age of parity. While epidemiological studies show a lifetime protective effect of breastfeeding, a temporary elevated risk of cancer development is reported in postpartum parents, which has been largely attributed to postpartum involution [131–133]. Age and parity greatly influence the risk of developing breast cancer, with an extended elevated risk of over 10 years in some cohorts, including multiparous parents and those having their first child after the age of 35 [133, 134]. Postpartum breast cancer is often aggressive, and the prognosis is poor with an additional risk of metastasis, highlighting the need for elevated monitoring during this time [135, 136]. Recent advances in transcriptomic profiling of milk-derived mammary epithelial cells suggest a novel non-invasive approach for identifying pathological changes in the mammary gland during lactation [115]. A recent study demonstrated that cell free tumor DNA can be detected in breast milk samples, highlighting the potential of breast milk as a novel non-invasive method for early cancer detection in this vulnerable population [137].

**Fig. 4:**
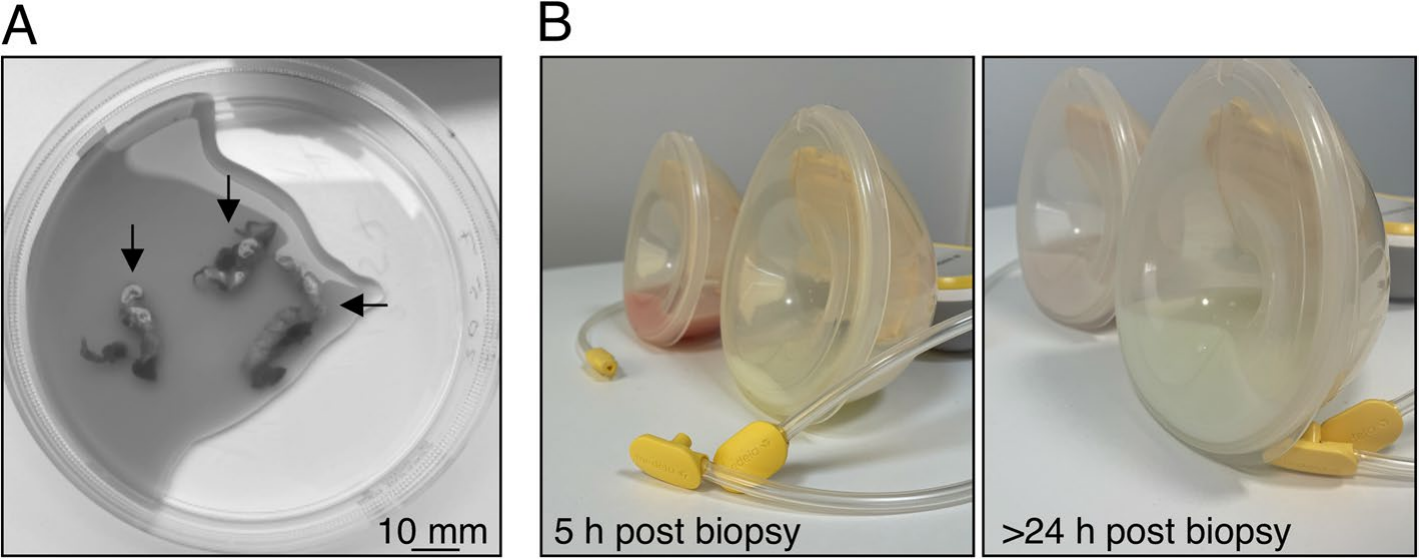
**A** Tissue obtained from a voluntary 6-core breast biopsy of a lactating parent (3 cores visible, black arrows), showing the amount of tissue that can be obtained using this approach. **B** In this instance, milk pumped from the biopsied (left) breast remained contaminated by blood for more than 24-h post-biopsy, which is considered normal

The low availability of donor tissue obtained from breastfeeding parents has unquestionably limited our understanding of the cellular and molecular physiology of human lactation. Rare biobanks exist, most notably the Susan G. Komen Tissue Bank at the IU Simon Cancer Center [12], with judicious use of these precious samples providing some opportunity for discoveries in mice to be tested in human. Tissue donation during this sensitive stage, however, is taxing (Fig. 4) and risks the development of milk fistulas or breast infection, both of which may compromise subsequent breastfeeding ability [138]. Moreover, tissue obtained from breastfeeding donors is often fixed or snap frozen, prohibiting its use in live cell/tissue assays. The elucidation of many aspects of cell and tissue dynamics during lactation thus requires input from mammalian model organisms, the most common of which is mouse, but includes domestic and agricultural animals. Improved methods for minimally-invasive, low risk longitudinal sampling of bovine tissue offers an important avenue for studies requiring repeat sampling of mammary tissue [139]. Protocols describing the creation of organoids that model aspects of lactation and involution have also now emerged and offer opportunities for assessing cellular dynamics during this stage [140].

Our understanding of the roles and mechanisms of the neurohormone oxytocin (OXT) during lactation is one example of an area of research that has benefited greatly by the use of animal models [141]. While connections between OXT and lactation were first observed in the early 1900 s, it was not until nearly a century later that this link was confirmed with the development of ligand and receptor null mice [142]. Knockout animals were reported to have normal parturition (an observation which has since been disputed [143]) but were unable to expel sufficient milk to support their litters [142, 144, 145]. More recently, advanced imaging studies using tissue obtained from transgenic mice have provided further depth to our understanding of OXT signaling in the mammary gland, with in vivo imaging of fluorescent reporter mice suggesting OXT-mediated basal cell contractions facilitate the release of mature lipid droplets from luminal cells [146]. Imaging of lactating tissue expressing genetically-encoded calcium (Ca^2+^) sensors has also revealed that basal cell contractions are Ca^2+^ signal dependent [147] and knockout models have shown a requirement for ORAI1 Ca^2+^ channels [148]. It is hard to imagine that these important mechanistic insights into the physiology of lactation could have been made without the use of mouse models.

Clinical and fundamental research in lactation biology go hand-in-hand, each having its enduring strengths and limitations. With the necessary funding, collaboration and communication, advances in transcriptomic screening could one day be harnessed to routinely test a consenting birthing parent for known mutations that may impact their ability to produce or release milk. This would help to reduce the distress that many new parents experience when they continue to struggle and ultimately fail to produce enough milk to support their baby [149]. Similarly, studies assessing genetic differences in cells obtained from the milk of parents with low milk supply or altered nutritional composition could help to identify mechanisms impacting lactation competency [150]. A greater understanding of the unique properties of breast milk may help start-up companies seeking to create breast milk in vitro as an alternative to infant formula [151]. Finally, a persistent clinical challenge in lactation biology is the deficit of galactagogues, with currently available options suffering from limited efficacy and, in some cases, significant potential adverse effects [152, 153]. A greater understanding of the signaling pathways underpinning lactation would surely aid in the discovery and development of new therapeutic agents to boost milk supply [154].

## Post-lactational involution

Upon weaning, the mammary gland undergoes coordinated tissue remodeling, a process known as post-lactational involution. This involves extensive epithelial cell death and stromal adaptations, which ultimately restore the gland to a state reminiscent of pre-pregnancy [155]. As this process varies across segments of the breast and by the specific schedule of weaning, studies in humans have proven especially difficult [46, 156, 157]. This is further complicated by limited access to normal human samples with comparable donor histories. To overcome some of these challenges, studies in mice have been particularly useful to help illuminate the cellular mechanisms underpinning post-lactational involution. The following section briefly discusses key lessons learned from mice, with further detail summarized in dedicated reviews [155, 158].

Conventional, synchronized involutional models in rodents are achieved by forcibly removing pups from the dam after lactation is fully established [159]. With this approach, two stages of post-lactational involution have been characterized [160, 161]. The first phase, driven by weaning-induced milk stasis, causes pronounced alveolar distention [162]. Remarkably, the changes that occur during this phase can be reversed if pups are re-introduced within 48-h, although this window is considerably longer in other species [163]. Beyond this, alveolar structures collapse and extensive cell death ensues, causing irreversible gland remodeling [159–161]. A large study, using over 100 tissue biopsies obtained from healthy post-lactational donors, has demonstrated that similar glandular remodeling programs occur in humans. The study authors found that by three months “post-weaning”, human breast composition was statistically indistinguishable from nulliparous comparators, when binned and scored on histological criteria. Levels of the milk lipid droplet surface marker adipophilin (perilipin 2) were also reduced by this stage, as were the presence of CD45 + immune cells [157].

One notable mechanistic discovery obtained from the interrogation of mouse mammary tissue during post-lactational involution is the importance of lysosomal mediated programmed cell death in this process. Studies exploring mouse mammary gland involution have demonstrated that luminal cells undergo a switch from a functional state characterized by the release of milk (during lactation) to a state marked by the re-uptake of milk (during involution), a process linked to levels of STAT3 phosphorylation in these cells [164, 165]. Endocytosed milk fat droplets were shown to be digested by lysosomes, resulting in lysosomal-mediated programmed cell death, likely due to the cytotoxic concentrations of free fatty acids in the cytosol [165]. In mice, the concentration of Ca^2+^ in milk is several hundred thousand-fold higher than that of the cytosol and it is also likely to have a death-inducing effect if other milk components are released into the cytoplasm through this mechanism [20, 166]. While lysosomal digestion of milk droplets may increase intracellular Ca^2+^ concentrations, downregulation of the apical Ca^2+^ pump PMCA2 in early involution has also been linked to the destruction of luminal cells through the lysosomal pathway [167, 168].

In mice, epithelial cell death is closely coordinated with changes in stromal composition, principally immune cell infiltration and re-expansion of the white adipose tissue. The “re-emergence” of mammary adipocytes to levels akin to that in pre-pregnancy, where they are a dominant cell type by area, has recently been shown to occur not by adipogenesis but by hypertrophy of existing adipocytes through the uptake of milk-derived lipids [169]. On the immune front, an increased immune cell requirement, particularly tissue macrophages, facilitates tissue remodeling in later stages of involution through the clearance of dead cells and cellular debris [28, 170–172]. Similar immune infiltration has been identified in the early involuting breasts of humans [156, 170]. A subset of macrophages have also been shown to associate with lymphatic vessels in mice, forming chimeric vessels – termed “macphatics” – and are thought to enhance neo-lymphangiogenesis [173].

Remodeling of the mammary microenvironment is necessary for completing post-lactational involution, however, the inflammatory environment of the involuting mammary gland may increase one’s cancer risk [133, 157]. The mechanistic link between windows of post-lactational involution and mammary cancer has been established in mice, where injection of cancerous cells into the early involuting glands of immunocompetent animals caused tumors to grow more rapidly cf. nulliparous controls [174]. Furthermore, studies in mice have offered insights as to how the weaning time and schedule may influence the inflammatory state and in turn the gland’s susceptibility to tumor progression [175, 176]. These studies demonstrated that glands of mice undergoing “abrupt” weaning (defined by a relatively short period of lactation and the sudden removal of pups) were histologically and molecularly different to the glands of mice undergoing “gradual” weaning (removal of pups between days 28–31 post-partum). This included differences in the number of F4/80 +, CD45 + and CD3 + cells on day 56 postpartum [176]. These studies suggest that gradual weaning might promote a less inflammatory and cancer-prone microenvironment, consistent with some epidemiological studies indicating a correlation between breastfeeding duration and breast cancer risk [177]. They pose important questions for cases of early lactation suppression (including in stillbirth and perinatal loss) and for the off-label use of drugs to suppress lactation (e.g., cabergoline). They also complement studies supporting roles for non-steroidal anti-inflammatory drugs (NSAIDs) in the post-partum period [157].

While weaning initiates complex remodeling that in some cases can promote pathogenic events, the typical post-involutional gland is in many aspects remarkably similar to the nulliparous state [156, 157, 178]. Studies in mice have, however, revealed lasting molecular changes of the post-involutional mammary gland [179, 180]. The luminal progenitor cells, in particular, have a changed transcriptional profile, favoring gene expression associated with pregnancy and lactation [179]. These findings support the concept of a 'lactational memory' within the mammary epithelium, that might prime the gland for subsequential pregnancies [180]. Evidence from mice has also shown enhanced alveologenesis in parous dams, enabling increased milk yield and rapid weight gain from the second litter onwards [180, 181]. Similarly, observational studies in humans have reported an increase in milk yield for some multiparous breastfeeding parents [182], suggesting that parity and prior breastfeeding may also increase the likelihood of earlier establishment of exclusive breastfeeding and breastfeeding success [183, 184]. However, previous breastfeeding experiences may influence a parous parent’s intentions to initiate breastfeeding [185, 186], and it is difficult to untangle whether breastfeeding outcomes in parous parents are influenced more by physiological ability or by perceived competence. In sum, while studies using parous human tissues are becoming more common and are important, samples are limited and contain natural variability. Due to our well documented understanding of the progression of mammary gland involution in mice and its parallels to human tissue, it is our view and the view of others [187] that studies in mice remain important and relevant for foundational research. We refer to Ref [187], which highlights the state of the field and important outstanding questions.

## Ageing and menopause

Around half of the global population experience the transition into menopause, typically as a consequence of age-related depletion of ovarian follicles [188, 189]. This process causes a gradual decline of ovarian sex hormones, particularly estrogen [190], triggering a spectrum of symptoms and physiological changes that differ in timing and severity across individuals [191, 192]. Despite its impact on quality-of-life and disease risks and sequalae, menopause remains a significantly underfunded area of research [193, 194].

The hormonal shift of menopause is also not well understood from mammary gland perspective [195, 196]. With increasing age, the mammary gland enters a stage called ‘age-related lobular involution’, which is distinct from post-lactational involution [46, 197]. During this stage, regression of the mammary epithelium, stromal remodeling, and expansion of adipocytes [197–199] collectively decrease breast density [200, 201]. However, a proportion of postmenopausal individuals retain high breast density, placing them at higher risk of developing breast cancer [202]. A fundamental understanding of the cellular heterogeneity of the aged mammary gland may help develop new strategies for the early detection of breast cancer. This could especially impact cases where high breast density delays early diagnosis through mammography [202, 203].

Recent single-cell and spatial transcriptomics studies have elucidated the cellular distribution of epithelial, stromal and immune cells within the aged mammary gland [204–206]. Interestingly, changes in epithelial expression and/or abundance have been observed in human and mouse studies, the majority of which appear species specific but which are not directly comparable due to differing study methods [207–209]. Nevertheless, these changes suggest age-related alterations of the mammary gland, the exploration of which may help in the understanding of aging associated cancer risk [209]. Despite advances in single-cell technologies [204], however, a comprehensive understanding of the postmenopausal gland remains challenged by the vast, accumulated, biological heterogeneity amongst individuals. Exposures such as parity; breastfeeding; age of menarche and menopause; hormonal treatments; and genetic predispositions influence the landscape of the individual aged mammary microenvironment [210–212].

Mouse models offer the field the advantage of greater control of genetics, environment and hormonal exposures. However, murine aging models have been debated in their relevance for human translational research, mainly because they do not seem to undergo definitive reproductive senescence like humans (reviewed in [193, 213]). Instead, mice exhibit prolonged estrus cycles with age and can enter stages of reversable reproductive pauses, termed estrus-pauses [193, 214, 215]. Initially, modeling a menopause-like state in mice has been done with surgical removal of ovaries in ovariectomy (OVX) models [216]. However, this model causes sudden ablation of ovarian hormones, more comparable to surgically-induced menopause than age-related menopause in humans [215]. The OVX model could provide insights into surgically-induced lobular involution for individuals undergoing bilateral oophorectomy for gender affirmation reasons or for pathological conditions (i.e. endometriosis, polycystic ovary syndrome or ovarian cancer). More recently, the VCD (4-vinylcyclohexene diepoxide) mouse model has shown promise to elucidate the menopausal transitional stage, termed perimenopause [217, 218]. Daily administration of the ovotoxin VCD causes a progressive ablation of ovarian follicles which induces a dwindling of ovarian hormones, similar to the perimenopausal state in humans [217]. This model has recently been characterized for studying the perimenopausal and menopausal mammary gland [219]. The VCD model could also be suitable to elucidate lobular involution in cases of premature ovarian failure, which has been linked to a heightened risk of reproductive cancers, including breast cancer [220, 221].

Collectively murine models remain indispensable for investigating how lobular involution following premature, surgical or age-related menopause affects mammary gland biology and implications for breast cancer progression. Greater funding is urgently needed in this area of research.

## Discussion

A greater understanding of mammary development and physiology—regardless of whether it is obtained from humans or mice—can have both knowledge- and translational- impacts across multiple fields of research. For the field of breast cancer research, for example, an improved understanding of the cells that can give rise to cancer, their dynamic interactions and mechanisms of proliferation and death will undoubtedly help with the design and development of new cancer treatments [11]. Similarly, the isolation of cells or cell free tumor DNA from breast milk could provide a low-cost, non-invasive method for detection of breast cancers [115, 137], and an understanding of stem cell dynamics may help with tissue regeneration following mastectomy [222]. A recent study has also demonstrated that the estrus/menstrual cycle influences sensitivity to neoadjuvant chemotherapy, an observation that began in mice and which has the potential to be rapidly translated to low-cost, improved patient care after confirmation in clinical cohorts [223]. Consideration too can be directed towards the impact of continued underfunding of foundational and clinical research in breast biology and women’s health, and the communication of this research to patients and carers. It has been estimated that more than 340 billion US dollars is lost globally each year due to inadequate breastfeeding knowledge and support [224]. Funding women’s health research and breast biology is an important investment.

There remain many criticisms of the use of mouse models in biomedical research. By some, mice are blamed for the reproducibility/replicability “crisis” in the scientific literature [225, 226]. Indeed, in some fields of research, the translational utility of mice is limited by important interspecies differences [225, 227] or the widespread availability of alternative and perhaps superior systems for investigation [228]. Here, we state that this is not the case for many fields of mammary gland research and provide strong arguments for the continued use of mice in thoughtfully designed and carefully described studies, where key variables such as strain background, estrus staging, tamoxifen dosing, sample handling etc. are well described and in line with leading protocols and available technical resources. Mouse models can also be dismissed as “easy”, “cheap” and/or “fast” alternatives to work that should be done in humans, the implication being that scientists that use these models are cutting corners or that the use of one system over another is superior, regardless of the methods and scope of the studies in question. This is simply not the case in mammary gland research, where a relatively basic study to examine the effect of gene knockdown in luminal cells in lactation (e.g., using *Wap*- or *Blg-Cre*), requires at least four to six months of crossbreeding and two subsequent rounds of pregnancy in the experimental animals [33]. This study also necessitates simultaneous breeding of wildtype mice for litter standardization, meaning that to generate a single knockout-control pair consumes approx. 40 animal lives. This is particularly problematic when larger cohorts need to be bred to account for unexpected losses e.g., due to problems with mating, littering (dystocia) and cannibalization. The situation can be even more difficult in other model organisms, e.g., naked mole rats, where enclosures need to closely mimic their natural subterranean habitat [229]. One criticism of mouse models, which is particularly relevant to the mammary field, however, is the dominance of tamoxifen-dependent systems for cre recombination. As a selective estrogen receptor modulator (SERM), tamoxifen affects normal mammary gland development [33] and should always be used with caution and at the lowest possible dose.

Recent methodological advances are enabling us to gain more insights with less sample. Single cell technologies, including single-cell RNA sequencing, epigenetics, DNA sequencing and proteomics are some examples where large volumes of data can be obtained from limited amounts of precious sample [230]. These techniques, however, can have different collection and processing requirements (e.g., the requirement for rapid tissue dissociation after excision), which can be hard to standardize in sterile operating theatres mid-surgery [231]. Recognizing this limitation, an elegant study out of Indiana University, utilizing fresh tissue biopsies donated through the Susan G Komen Tissue Bank, identified 13 different human epithelial cell types in the normal breast [230]. Of note, these authors have previously demonstrated, also using breast biopsies from this tissue bank, that although tissue acquired from mammary reduction procedures is often labelled “normal”, they are in fact histologically and molecularly abnormal and thus also have limits on their translatability. Finally, although an extremely valuable resource, it is important to acknowledge that when only a small number of biobanks exist (typically associated with universities and hospitals in the Global North) a limitation is placed on donor diversity, and thus studies utilizing these samples may also have problems of translatability. Finally, the emergence of single cell studies necessitates dialogue and consensus on breast cell types and their nomenclature, which has recently been proposed and published [42].

In sum, it is often unnecessary and unreasonable for many investigations in mammary gland research to be fully or partly replicated in human samples by the study authors. The availability of truly normal human samples (with appropriate handling/storage for the specific downstream application); the historic and continued neglect of the fields of women’s health and female biology; issues related to the long-term propagation of mammary epithelial cells in culture; and the considerable similarities between mouse and human mammary development and physiology, make mouse models relevant and important both now and into the foreseeable future. Needless to say, mice aren’t just “little women”, however, when they are used thoughtfully and with an appropriate discussion of their limitations, they can still have a big impact on the field of mammary gland research.

## Data Availability

No datasets were generated or analysed during the current study.
